# Pedigree analysis of Czech Holstein calves with schistosoma reflexum

**DOI:** 10.1186/1751-0147-54-22

**Published:** 2012-04-02

**Authors:** Jindrich Citek

**Affiliations:** 1 Department of Genetics and Animal Breeding, Faculty of Agriculture, South Bohemia University, Studentska 13, Ceske Budejovice CZ37005, Czech Republic

**Keywords:** Cattle, Schistosoma reflexum, Inheritance, Congenital, Malformation

## Abstract

**Background:**

Schistosoma reflexum (SR) is congenital syndrome briefly characterized by visceral eventration, severe dorsoflexion and ankylosis of the spine and arthrogryposis. A genetic etiology has been proposed, but conclusive evidence has not yet been provided.

**Methods:**

Pedigree analysis was carried out in 29 cases of SR in Czech Holsteins and Holstein crosses. Genetic relationship was evaluated and inbreeding coefficients calculated. Pedigrees of 15 Czech Holsteins fathering non-SR affected calves were used for comparison.

**Results:**

Twenty-one cases occurred in one pedigree founded by three sires while three SR calves occurred in another pedigree with a common grandfather. The sex ratio between affected males and females was 11:6. Affected calves shared common ancestors different from those shared by the unaffected calves. The inbreeding coefficient in the SR affected calves was not increased compared to unaffected calves.

**Conclusions:**

The findings are consistent with SR being inherited autosomal recessively. Further studies are however needed to confirm this and therefore a breeding trial is recommended where a suspected heterozygous sire is mated to closely related females.

## Background

Schistosoma reflexum (SR) is congenital syndrome briefly characterized by severe abdominal fissure with total eventration of viscera, marked dorsoflexion, and ankylosis of the spine and limbs. It occurs in all food animals, but is most common in cattle. SR usually requires assisted delivery, in most cases Caesarean section or foetotomy [1,2]. Although SR is rare, the high risk of dystocia in such cases is a welfare problem and the required veterinary assistance has a negative economic impact on farm economy.

Most studies of SR have dealt with the morphology of the syndrome [2-4], although some authors have also proposed a genetic background [5]. However, conclusive evidence has not yet been provided.

This study was performed to further evaluate a possible genetic aetiology through pedigree analysis of multiple SR cases in Czech Holstein cattle.

## Methods

Twenty-nine cases of SR diagnosed and reported by veterinary surgeons to the Czech surveillance program for bovine genetic disorders were included. The study was limited to Holstein and Holstein-crossbred calves fathered by sires born between January 1986 and December 2001. The sires were born in the Czech Republic or included in the herd book due to semen import. Parentage control was not performed to confirm registered descent. The cases occurred on farms spread across the Czech Republic. Six cases were females, 11 were males while the sex was not recorded in 12 cases.

The pedigree of each case was examined for common maternal and paternal ancestors (inbreeding loops) and for ancestors shared by other cases. When different cases shared a common ancestor combined pedigrees were constructed.

Wright's inbreeding coefficient (Fx) was calculated when a common maternal and paternal ancestor was found by the equation:

$$\text{Fx}=\Sigma \;0.5{\;}^{{\text{n}}_{\text{1}}+{\text{n}}_{2}+1}$$

Where *n_1 _*and *n_2 _*are the number of generations between the SR affected calf and the common ancestor in the paternal and maternal part of the pedigree, respectively.

Pedigrees of 15 SR unaffected Holstein calves being progeny of sires that had not registered SR affected progeny and born in the same period as sires having registered SR affected progeny were sampled from the Plemdat database [6] at random. These sires were used as controls, i.e. their pedigrees were evaluated for the presence of sires genetically associated with SR affected calves and their inbreeding coefficient was calculated.

## Results

Although the diagnosis of SR was based on field observations by a number of veterinary surgeons, the diagnoses are considered reliable as the morphology of SR is probably well known to veterinarians. Detailed morphological descriptions were not available, although additional defects as e.g. fissure of the genitals and polypodia were reported.

Pedigree analyses included seven generations in most cases, while nine generations pedigrees were available in some cases. In a number of cases, pedigrees were incomplete due to unregistered descent. One case was omitted from the calculations as the descent of dam of an affected calf was unknown. The 29 SR cases were progeny of 29 sires.

**Figure 1:**
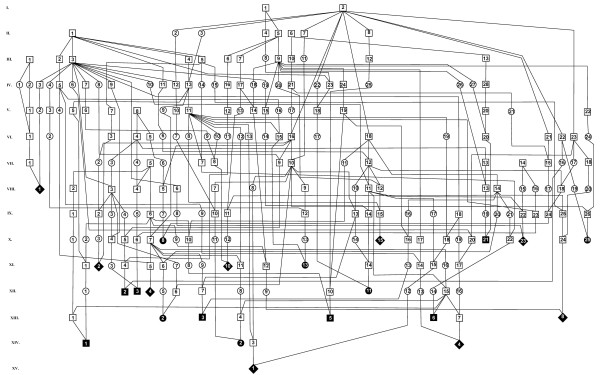
**Pedigree of 21 schistosoma reflexum affected calves**. The animals in Figure 2 are not included here. Generations are numbered from the top of the pedigree in uppercase Roman numerals. Individuals in each generation are numbered from the left in Arabic numerals. Symbols indicate: □/■: Unaffected/affected male; ○/●: Unaffected/affected female; ◊/♦: Unaffected/affected individual of unknown sex.

**Figure 2:**
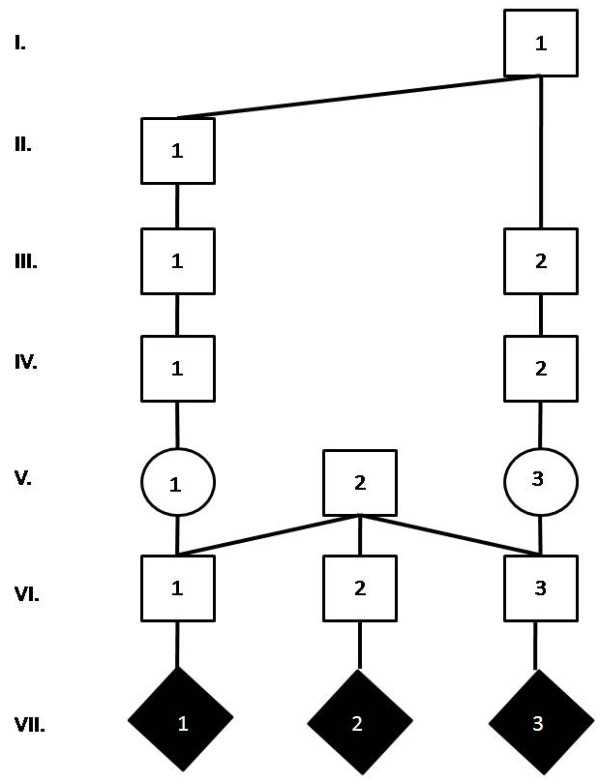
**Pedigree of three schistosoma reflexum affected calves**. The common grandfather V/2 is crossbred Holstein (75%) and Simmental (25%). Sires VI/1 and VI/3 are crossbred Simmental (63%) and Holstein (37%). Sire VI/2 is crossbred Simmental (51%) and Holstein (49%). Generations are numbered from the top of the pedigree in uppercase Roman numerals. Individuals in each generation are numbered from the left in Arabic numerals. Symbols indicate: □/■: Unaffected/affected male; ○/●: Unaffected/affected female; ◊/♦: Unaffected/affected individual of unknown sex.

Twenty-one SR affected calves were genetically related (Figure 1). Of these, 18 SR cases had a common ancestor in the paternal and maternal pedigrees (inbreeding loops). All sires producing SR cases were purebred Holsteins. Overall 106 ancestors with multiple occurrences were identified by analysis of the individual SR pedigrees. Usually the number of repetitions ranged from two (48 ancestors) to seven (six ancestors). However, three sires (I/2, II/1 and III/2 in Figure 1) were repeated 61, 58 and 22 times, respectively. In addition to the pedigree including 21 cases, a minor pedigree with a few cases of SR were identified (Figure 2). Three cases of SR were progeny of the same grandfather, and two of the grandmothers were related while the third was of unknown descent.

For comparison, 15 additional sires not fathering SR affected calves were analysed. These 15 sires were not of the same lines as the sires fathering SR calves. Of the three sires (I/2, II/1 and III/2) often present in the pedigrees of SR calves (Figure 1), solely I/2 occurred in these comparative pedigrees but only rarely. Other ancestors with repeated occurrence in SR pedigrees hardly appeared in the comparative pedigrees thus indicating that affected calves shared common ancestors different from those shared by the unaffected offspring.

The inbreeding coefficient was calculated for SR calves and the sires selected for comparison. The coefficient was calculated only for calves with common ancestors up to and including the 5^th ^generation in the parental pedigrees. For SR calves, only 11 individuals complied with this requirement. All 11 calves were included in the pedigree shown in Figure 1, and they had an average Fx of 0.94% (range: 0.20% to 2.34%). The average inbreeding coefficient of the sires fathering SR calves was 1.55%. The Fx of control Holstein sires not having registered SR affected offspring was 0.03%, while the average coefficient of all sires in the Czech registry was 0.67%. If only registered sires with a coefficient greater than zero were included, the average inbreeding coefficient was 2.49%.

During the period 1986-2001, 2740 Holstein sires entered the Holstein pedigree herd book in the Czech Republic. Thus, 0.84% of Holstein sires fathered a SR calf.

## Discussion

Analysis of pedigree data showed that 24 out of 29 registered SR cases occurred in two breeding lines (Figures 1 and 2). Within the large pedigree (Figure 1), some animals as e.g. sire XII/15, were genetically closely related to several SR cases. It should be clearly emphasized, that in a large complicated pedigree such as that in Figure 1, the common ancestors may occur by chance, especially if they have been widely used. This is the case for I/1 (Osborndale Ivanhoe, born 1952, US1189870), I/2 (Round Oak Rag Apple Elevation, born 1965, US1491007), II/1 (Pawnee Farm Arlinda Chief, born 1962, US1427381) and III/2 (S-W-D Valiant, born 1973, US1650414) that are important breeding sires used worldwide in Holstein cattle breeding. Sires as these will by chance occur the pedigree of defective Holstein calves [7]. However, the occurrence of sires such as XII/15 (Figure 1) and V/2 (Figure 2) related to several cases of SR may indicate a genetic aetiology.

In addition to the 29 SR cases included in this study, six other SR cases were registered (data not shown). Five of these were purebred Czech Simmentals or Simmentals crossbred with Ayrshire or a beef breed, while one case was of unknown descent. These findings show that SR is not restricted to the Holstein breed in the Czech Republic.

Although not all cases of SR occurred in a familial pattern, the findings of sires genetically associated with multiple cases indicate that the SR is inherited. Familial occurrence of SR cases has been reported previously [8]. Inbreeding loops might have been recognised in all cases if sufficient information on ancestors had been available. But due to the structure of cattle breeding, common ancestors and thus inbreeding loops would probably have been found by chance and therefore not provided any clear evidence for the inheritance. Studies of multiple closely related cases by molecular techniques such as SNP-based association mapping [9] would probably be most helpful in demonstrating a genetic aetiology. To ensure the accurate diagnosis that is needed to obtain a uniform collection of SR cases for molecular examination, further research should be carried out to establish the morphology of SR cases in detail. It can not be neglected that different mutations may be associated with a SR phenotype.

Based on the present results and those of others [3,5], cases of SR seems to occur in patterns consistent with autosomal recessive inheritance. X-linked recessive inheritance seems unlikely as affected males and females occur after breeding between unaffected animals and dominant inheritance is not likely as ancestors most likely were unaffected by this lethal syndrome [3]. Also, a non-genetic aetiology seems unlikely as cases occurred as isolated incidents and as cases of SR occur in twins with only one progeny being affected [10-14].

To confirm the genetic aetiology of SR and to establish a collection of cases for molecular investigations, a breeding trial is recommended where a sire that have had a SR affected offspring is mated to closely related females, e.g. some of his own daughters. Until then it is recommended to use such sires with caution to avoid unintended spread of deleterious genes in the population.

## Conclusions

The findings are consistent with SR being inherited autosomal recessively. Further studies are however needed to confirm this and therefore a breeding trial is recommended where a suspected heterozygous sire is mated to closely related females.

## Competing interests

The author declares that they have no competing interests.

## Authors' contributions

JC made the design of the study. He found the common ancestors, made the common pedigrees, evaluated the control pedigrees, inbreeding coefficients, wrote the manuscript, and formulated conclusions. The author read and approved the final manuscript.
